# Mucosal impedance as a diagnostic tool for gastroesophageal reflux disease: an update for clinicians

**DOI:** 10.1093/dote/doae037

**Published:** 2024-04-26

**Authors:** Matthew Marshall-Webb, Jennifer C Myers, David I Watson, Tim Bright, Taher I Omari, Sarah K Thompson

**Affiliations:** Discipline of Surgery, College of Medicine and Public Health, Flinders University, Adelaide, SA, Australia; Discipline of Surgery, College of Medicine and Public Health, Flinders University, Adelaide, SA, Australia; Department of Surgery, The University of Adelaide, Adelaide, SA, Australia; Discipline of Surgery, College of Medicine and Public Health, Flinders University, Adelaide, SA, Australia; Discipline of Surgery, College of Medicine and Public Health, Flinders University, Adelaide, SA, Australia; Human Physiology and Centre for Neuroscience, College of Medicine and Public Health, Flinders University, Adelaide, SA, Australia; Discipline of Surgery, College of Medicine and Public Health, Flinders University, Adelaide, SA, Australia

**Keywords:** esophageal, gastroesophageal reflux (GERD), impedance, pH-impedance

## Abstract

Mucosal impedance is a marker of esophageal mucosal integrity and a novel technique for assessing esophageal function and pathology. This article highlights its development and clinical application for gastroesophageal reflux disease (GERD), Barrett’s esophagus, and eosinophilic esophagitis. A narrative review of key publications describing the development and use of mucosal impedance in clinical practice was conducted. A low mean nocturnal baseline impedance (MNBI) has been shown to be an independent predictor of response to anti-reflux therapy. MNBI predicts medication-responsive heartburn better than distal esophageal acid exposure time. Patients with equivocal evidence of GERD using conventional methods, with a low MNBI, had an improvement in symptoms following the initiation of PPI therapy compared to those with a normal MNBI. A similar trend was seen in a post fundoplication cohort. Strong clinical utility for the use of mucosal impedance in assessing eosinophilic esophagitis has been repeatedly demonstrated; however, there is minimal direction for application in Barrett’s esophagus. The authors conclude that mucosal impedance has potential clinical utility for the assessment and diagnosis of GERD, particularly when conventional investigations have yielded equivocal results.

## INTRODUCTION

Mucosal impedance has emerged as a new parameter for assessing esophageal function and pathology. Mucosal impedance is a marker of mucosal integrity.^1^ It can differentiate patients with gastroesophageal reflux disease (GERD) from normal controls and may identify which patients will respond to therapy.^1^^,^^2^ Mucosal impedance can also be used to assess eosinophilic esophagitis (EoE) and Barrett’s esophagus.^3^ This article summarizes the development of technology to assess mucosal impedance and discusses its clinical utility for the diagnosis of common upper gastrointestinal conditions.

### What is esophageal luminal impedance?

**Fig. 1:**
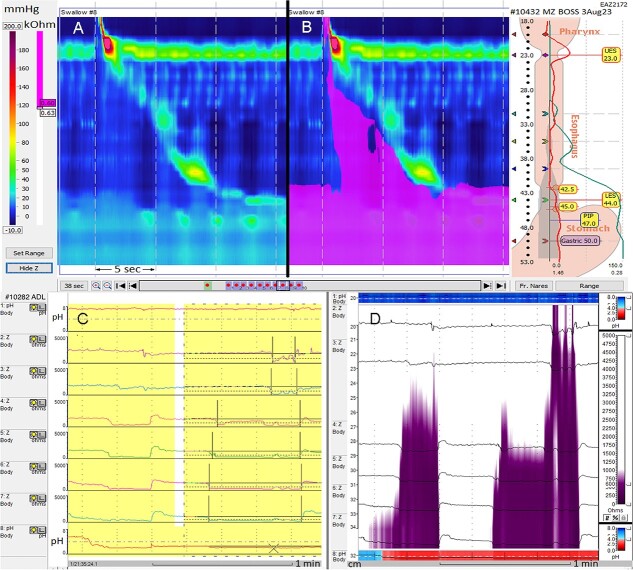
High-resolution impedance-manometry (36 pressure; 18 impedance) of 5 mL liquid swallow (SBM-0): pressure contour plot (**A**, LHS view) and impedance overlay (**B**, RHS view) demonstrating bolus movement in relation to the esophageal lumen contractions. Multichannel impedance-dual pH (6 impedance; 2 pH) showing two esophageal reflux events by proximal extent (impedance) and acidity (pH) in line plot mode (**C**, LHS view) and color plot mode (**D**, RHS view) [impedance at 3, 5, 7, 9, 15, 17 cm; pH at 5, 20 cm above proximal LES margin; note pH data displayed at the top and bottom of plots C and D]. (Images courtesy Dr J Myers, Oesophageal Function, Adelaide, Australia.)

In the esophagus, multi-channel intraluminal impedance (MII) refers to multiple electrodes spaced along the length of a catheter placed in the esophageal lumen. It can detect the electrical impedance of low-electrical current between any two paired ring-electrodes when bridged by an electrically conductive medium (e.g. swallowed fluid or gastric fluid), indicating bolus movement (Fig. 1).^1^ This technology can be combined with: (i) manometry (MII-manometry) to assess the relationship of bolus movement with esophageal peristalsis or (ii) pH monitoring (MII-pH) to determine both the physical characteristics of the substance within the esophagus (i.e. liquid/mixed or gas by impedance; acidic reflux or non-acidic reflux by pH) and, in the case of reflux, the proximal extent of the reflux event (Fig. 1).

In clinical practice, impedance is most often used in conjunction with pH testing to detect fluid in the esophagus and thereby diagnose a patient with GERD based on abnormal reflux events. As impedance detects reflux over multiple impedance channels and is then defined as non-acidic (pH ≥7) or weakly acidic reflux (pH 4–7) events, it can complement pH monitoring to identify reflux events that would be missed by pH monitoring of acid reflux alone (pH <4). Impedance measures also demonstrate bolus transport through the length of the esophagus, which, when combined with high-resolution manometry, can provide supportive evidence of motility disorders.

### What is mucosal impedance?

Mucosal impedance refers to the baseline impedance of the esophageal mucosa. With the development of intraluminal impedance monitoring (ambulatory monitoring or direct mucosal contact during endoscopy), patients with GERD, EoE, and Barrett’s esophagus demonstrate a lower baseline impedance compared with healthy controls.^4^ Lower mucosal impedance indicates decreased mucosal integrity and therefore suggests mucosal damage or pathology.^1^ For example, dilated intercellular spaces and the associated disruption of tight cellular junctions allow leakage of ions, thereby decreasing mucosal impedance.^1^ A seminal paper by Kessing *et al.* demonstrated a significant inverse correlation between 24-hour distal esophageal acid exposure time (AET) and baseline luminal impedance (*P* < 0.001).^2^

### How is mucosal impedance calculated?

**Fig. 2:**
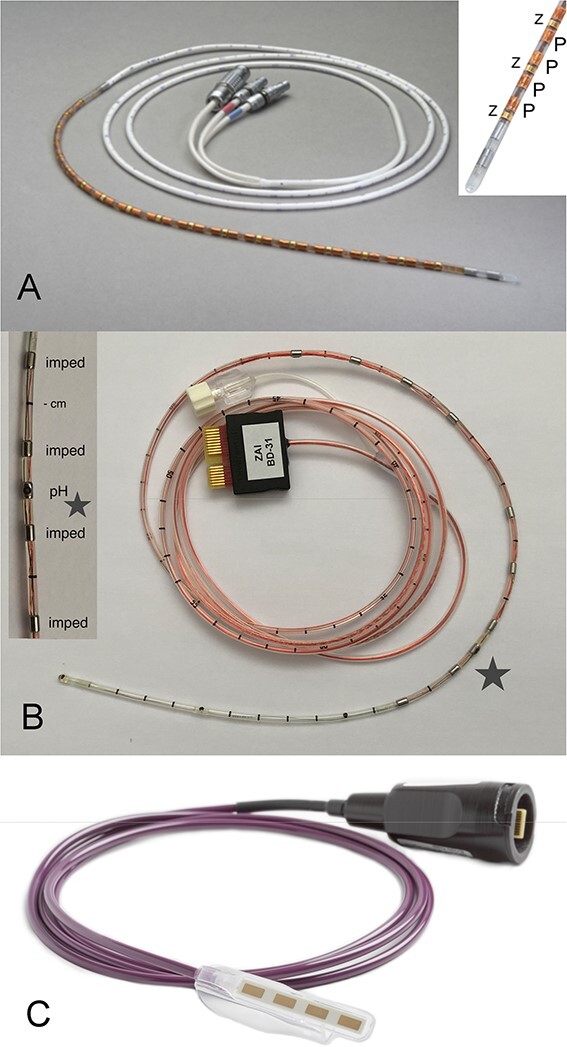
(**A**) Solid-state high-resolution impedance-manometry catheter (4.2 mm ∅, 36 pressure; 18 impedance, Medtronic EAZ). (Courtesy Medtronic Australasia Pty Ltd.) (**B**) Dual pH-impedance reflux catheter (2.3 mm ∅, 2 pH (15 cm apart); 6 impedance, Diversatek). (**C**) MiVu Endo Cap attaches to an endoscope for direct vision and targeted acquisition of real-time mucosal impedance, for display of mucosal integrity contour pattern. (Courtesy Diversatek Healthcare Inc. USA.)

There are three established methods for calculating mucosal impedance for the distal esophageal lumen (using technology shown in Fig. 2), each with strengths and weaknesses.

#### Mean nocturnal baseline impedance (MNBI)

MNBI is the most widely used and accepted method for assessing the mucosal impedance of the distal esophagus.^4^ It is determined from baseline impedance data recorded during an ambulatory 24-hour esophageal intraluminal pH impedance study. The direct apposition of the impedance sensors to the esophageal mucosa is crucial for this method. Mucosal apposition will vary with patient movement, swallowing, and reflux events. These confounders are minimized by taking measurements at night when the patient is sleeping. Baseline impedance readings are taken from an impedance sensor located in the distal esophagus (above the lower esophageal sphincter) at three separate time points, usually during a 10-minute period at 1, 2, and 3 a.m., and then averaged.^4^ As MNBI can be calculated using impedance data collected during routine diagnostic 24-hour pH impedance studies, no additional equipment or catheters are required.

While it is possible to calculate baseline impedance levels in the proximal esophagus, studies to date do not show any significant difference between GERD patients and healthy controls.^1^^,^^2^ However, MNBI measurements have been found to correlate with the severity of distal esophageal AET (pH <4) and can distinguish patients with functional heartburn from patients with a hypersensitive esophagus, non-erosive GERD, or erosive GERD.^5^^,^^6^ Frazzoni *et al.* reported a MNBI value of less than 1500 Ω in patients with conclusive GERD based on abnormal AET.^7^ The median MNBI value for healthy subjects with normal AET (irrespective of symptoms) was consistently above 2500 Ω.^7^^,^^8^

#### High-resolution impedance manometry (HRIM)

Baseline impedance is calculated from data recordings of high-resolution impedance manometry (HRIM). There are several different catheters available with varying but similar configurations of sensors. For example, a solid-state HRIM catheter may consist of 32–36 pressure sensors spaced 1 cm apart and 16–18 impedance sensors spaced 2 cm apart (Fig. 2). The baseline impedance values can be calculated between two of the impedance sensors in the distal esophagus (2–3 cm above the lower esophageal sphincter) during the ‘rest period’, after acclimatization and prior to the initiation of study bolus swallows. This method correlates with MNBI in terms of assessing mucosal impedance.^9^ However, HRIM provides easier and quicker access to mucosal integrity assessment because it can be calculated during the patient visit and does not require 24-hour reflux monitoring.

As a HRIM catheter is not inserted under vision, direct contact with the esophageal mucosa cannot be assured. Contractile segment impedance (CSI) was developed to overcome this problem, and during HRIM, it determines mucosal impedance while catheter sensors are pressed against the mucosa as the peristaltic contraction passes over them. Studies show CSI from HRIM correlates with MNBI on MII-pH and inversely correlates with pathologic AET from 24-hour pH-impedance monitoring, further enhancing the evaluation of GERD.^9^

#### Mucosal impedance probes (single channel and balloon)

Specialized mucosal impedance probes developed for use during endoscopy can be directly applied to the esophageal mucosa under vision. Different devices include: (i) through-the-scope probes with impedance sensors located on the probe tip (single channel) or (ii) impedance sensors on the outside of a small balloon, which is inflated in the distal esophagus to obtain mucosal contact.^3^ The current scope device enables mucosal impedance detection in real-time by direct apposition of four specialized sensors across the esophageal mucosa^4^ (Fig. 2).

The advantages of this technology are that specific regions of the esophageal mucosa can be targeted and assessed in real-time (in contrast to the other methods). However, this is a developing technology, and the probes are not widely available for clinical use [at the time of writing FDA approved; CE and TGA registration pending]. However, preliminary studies validate the use of these catheters in GERD and EoE.^3^

### Clinical relevance

#### Gastroesophageal reflux disease

Reflux disease is a common condition affecting 20–30% of the population.^3^ GERD is defined by the Lyon 2.0 consensus as ‘conclusive evidence of reflux related pathology on endoscopy and/or abnormal reflux monitoring in the presence of compatible troublesome symptoms’.^4^ Confirmation of GERD can be challenging as it is a complex disease with heterogeneous and often unreliable symptomatology. With the development of high-resolution manometry ± impedance and 24-hour ambulatory pH ± impedance testing, our understanding of the impact of this disease is greatly enhanced, especially when combined with symptom correlation or symptom indices.

The greatest potential application of mucosal impedance, by whichever method it is measured, is to confirm the diagnosis of GERD. MNBI values at the distal esophagus are known to be lower in patients with type 3 EGJ morphology (separation of the lower esophageal sphincter and crural diaphragm by >3 cm) and GERD-associated dysmotility.^10^ However, mucosal impedance may also have particular relevance to patients with more equivocal diagnostic findings. Given mucosal impedance is a surrogate marker for mucosal integrity; this allows clinicians to determine the degree of long-term mucosal damage due to chronic reflux, even in the absence of macroscopic changes such as esophagitis.^5^ Preliminary data shows mucosal impedance can discriminate patients with GERD, both erosive and non-erosive, from individuals without GERD with greater certainty and less ambiguity than other modalities.^3^^,^^5^ In addition, mucosal impedance values increase with mucosal healing, demonstrating an objective measure of treatment response.^11^

For patients with an equivocal pH study (AET 4–6%), or those with non-erosive GERD, a lower MNBI identifies which patients are more likely to respond to PPI, compared to patients who were refractory to PPI therapy. Rengarajan *et al.* demonstrate that for patients with an abnormal (>6%) or equivocal AET (4–6%), a low MNBI identifies patients who show improvement following anti-reflux treatment. Improvement of symptoms was greater in patients with low MNBI (<2292 Ω) following initiation of PPI compared to normal MNBI (>2292 Ω): 60.1% versus 17.4% (*P* < 0.005). A similar trend, although not statistically significant, was seen in a surgical cohort; 82.0% versus 53.8% (*P* = 0.06).^12^ However, the number of patients in the surgical cohort was small: 44 patients, compared to 308 patients in the medical cohort. Recently, Frazzoni *et al.* validated MNBI against the AET thresholds outlined in the original Lyon Consensus for diagnosing GERD. The MNBI threshold value of 2000 Ω had an odds ratio of 5.7 for the detection of patients with PPI-responsive non-erosive esophagitis, compared to an AET of 4%.^7^

An important potential advantage of mucosal impedance for patients is that MNBI can also predict treatment response in patients on or off PPI therapy.^11^^,^^13^ This is significant because cessation of PPI therapy can exacerbate symptoms. Frazzoni *et al*. examined 189 patients with PPI-refractory GERD and found MNBI was significantly lower in GERD patients (refractory esophagitis, healed esophagitis, and non-erosive esophagitis) compared to patients with functional heartburn.^11^ However, performing MII-pH testing on PPI therapy is still debated in the literature, and further studies are required.

The Lyon 2.0 consensus, considered a clear and practical guide for the modern diagnosis of reflux disease, refined esophageal testing parameters for the establishment of a diagnosis of GERD. MNBI is acknowledged as a useful adjunct in the diagnosis of GERD.^4^ The consensus highlights a multicentre study by Frazzoni *et al.*, which demonstrated that patients with conclusive GERD (abnormal AET) consistently had MNBI values <1500 Ω.^7^ Sifrim *et al.* examined a large worldwide cohort of healthy asymptomatic and symptomatic subjects with normal AET and found that MNBI values were consistently >2500 Ω.^8^

The important take-home message is: if there is conclusive evidence of GERD on endoscopic or pH/MII-pH testing, then mucosal impedance will not add to the diagnostic algorithm. However, if clinical criteria are not met for the diagnosis of pathological GERD, a low mucosal impedance in the distal esophagus may provide support for anti-reflux treatment.

#### Barrett’s esophagus

Studies show that mucosal impedance is reduced in Barrett’s esophagus, similar to patients with severe esophagitis.^14–16^ Kataria *et al.* examined the baseline distal esophageal mucosal impedance in 45 patients: 16 with Barrett’s esophagus, 19 with esophagitis, and 10 with healthy controls. They showed that baseline mucosal impedance at the first, second, and third sensors above the lower esophageal sphincter was reduced in Barrett’s esophagus patients (mean ± SEM: 1.37 ± 0.45, 0.97 ± 0.27, and 0.81 ± 0.20) compared to healthy volunteers (8.73 ± 0.60, 9.2 ± 0.73, and 6.94 ± 0.99) (*P* < 0.001). Baseline mucosal impedance was also reduced when compared to esophagitis patients; however, statistical significance was only seen at the second and third impedance sensors above the lower esophageal sphincter (*P* < 0.052).^14^ There was a strong correlation between the length of low impedance and the length of Barrett’s esophagus by *C* score, *M* score, and pathology (*P* < 0.015). However, this study was limited by its retrospective nature, small sample size, homogeneous control group (young and male), high percentage of patients with hiatus hernia in the Barrett’s esophagus and esophagitis groups, and an increased incidence of ineffective esophageal motility in the Barrett’s esophagus group (44% of patients).

Pilot data from our group also found that patients with Barrett’s esophagus had very low mucosal impedance (median 477 Ω), potentially distinguishing them from other patients with GERD (non-erosive = 873 Ω and erosive = 485 Ω) (*P* = 0.006).^17^ The diagnostic utility of mucosal impedance in Barrett’s esophagus warrants further investigation in future studies.

#### Eosinophilic esophagitis (EoE)

Mucosal impedance is also reduced in EoE.^18^^,^^19^ In particular, it allows for the assessment of the whole esophagus (i.e. non-invasive measurements at any location throughout the esophagus), compared to biopsies in only a few targeted locations. A cut-off of <2300 Ω can represent active rather than inactive EoE, with a 90% sensitivity and 91% specificity.^18^ However, mucosal impedance correlates poorly with peak eosinophil count.^18^

Mucosal impedance can also be a useful tool for distinguishing EoE from GERD. Patients with GERD will demonstrate an increase in mucosal impedance from the distal to the proximal esophagus. In contrast, mucosal impedance in EoE will be similar over the entire length of the esophagus, with no clear distal to proximal gradient.^19^^,^^20^

### Future directions

This update for clinicians outlines three exciting applications of this new technology in the diagnosis, treatment, and surveillance of reflux disease, Barrett’s esophagus, and EoE. More work is needed, and our group is currently running a prospective trial using HRIM in patients with Barrett’s esophagus, treated with either medical or surgical therapy for reflux control.

In addition, a recent publication by Rogers *et al.* describes the use of an artificial intelligence (AI) program to analyze ambulatory pH-impedance studies in response to the labor intensive manual analysis of pH-impedance studies.^21^ Surprisingly, AI could instantaneously identify and censor esophageal events with a sensitivity of 88.5%. There was a strong correlation between MNBI and AI calculated recumbent baseline impedance (*r*^2^ = 0.79, *P* < 0.001 at 3 cm and *r*^2^ = 0.86, *P* < 0.001) at 5 cm above the lower esophageal sphincter.^21^ This innovative use of AI will further promote the uptake of metrics like MNBI.

## CONCLUSION

Mucosal impedance is an emerging new application of technology and is a marker of esophageal mucosal integrity. The development of this technology continues to enhance the understanding and assessment of esophageal disease. Currently, the most useful application of mucosal impedance for clinicians is the assessment of GERD in patients, in which other investigations yield equivocal results.

## Notes

Specific author contributions: Matthew Marshall-Webb (Conceptualization, Data curation, Methodology, Writing—original draft), Jennifer Myers (Conceptualization, Methodology, Supervision, Writing—review & editing), David Watson (Supervision, Writing—review & editing), Tim Bright (Supervision, Writing—review & editing), and Taher Omari (Conceptualization, Methodology, Supervision, Writing—review & editing)

Financial support: None.
